# The global role of G6PD in infection and immunity

**DOI:** 10.3389/fimmu.2024.1393213

**Published:** 2024-06-13

**Authors:** Shivang S. Shah, Elizabeth F. Stone, Richard O. Francis, Matthew S. Karafin

**Affiliations:** ^1^ Department of Pediatrics, Columbia University, New York, NY, United States; ^2^ Department of Pathology and Cell Biology, Columbia University, New York, NY, United States; ^3^ Department of Pathology and Laboratory Medicine, University of North Carolina, Chapel Hill, NC, United States

**Keywords:** glucose-6-phosphate dehydrogenase, G6PD, immunity, immunometabolism, G6PD deficiency

## Abstract

Glucose-6-phosphate dehydrogenase (G6PD) deficiency is the most common enzymopathy in humans. G6PD is an essential enzyme in the pentose phosphate pathway (PPP), generating NADPH needed for cellular biosynthesis and reactive oxygen species (ROS) homeostasis, the latter especially key in red blood cells (RBCs). Beyond the RBC, there is emerging evidence that G6PD exerts an immunologic role by virtue of its functions in leukocyte oxidative metabolism and anabolic synthesis necessary for immune effector function. We review these here, and consider the global immunometabolic role of G6PD activity and G6PD deficiency in modulating inflammation and immunopathology.

## 1 Introduction

**Figure 1:**
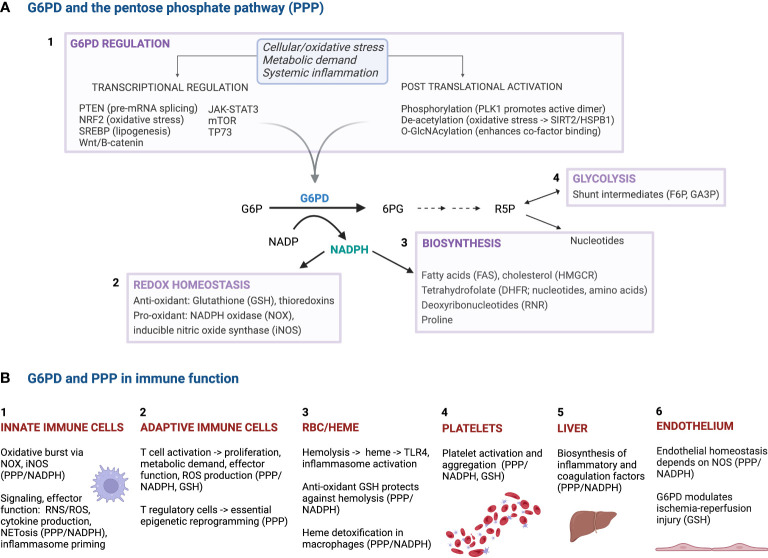
The roles of G6PD and the pentose phosphate pathway (PPP) in cellular and immune function. **(A)** The top panel highlights the multifaceted role of G6PD in cellular metabolism, including biosynthesis and the response to oxidative stress. In response to key triggers such as cellular/oxidative stress, metabolic demand and systemic inflammation, active G6PD production is upregulated by various factors/pathways at the transcriptional and post-transcriptional levels. The resultant NADPH produced is key in redox homeostasis and several biosynthetic functions, including lipid and nucleotide synthesis. **(B)** The bottom panel highlights the pleiotropic impact that G6PD and PPP activity have on immune function, through diverse impacts on biosynthesis and activation/effector phenotypes, redox and endothelial homeostasis, cell signaling and inflammasome priming, and mitigating RBC hemolytic stress. PTEN, phosphatase and tensin homolog; mRNA, messenger ribonucleic acid; NRF2, nuclear factor erythroid 2-related factor 2; SREBP, sterol regulatory element binding protein; JAK-STAT3, Janus kinase-signal transducer and activator of transcription 3; mTOR, mammalian target of rapamycin; TP73, tumor protein p73; PLK1, polo-like kinase 1; O-GlcNAc, O-linked β-N-acetylglucosamine; SIRT2, sirtuin 2; HSPB1, heat shock protein family B member 1; G6P, glucose 6-phosphate; G6PD, glucose 6-phosphate dehydrogenase; 6PG, 6-phosphogluconate; NADP, nicotinamide adenine dinucleotide phosphate; NADPH, reduced form of NADP; R5P, ribulose 5-phosphate; F6P, fructose 6-phosphate; GA3P, glyceraldehyde 3-phosphate; GSH, glutathione; TRX, thioredoxin reductase; FAS, fatty acid synthase; iNOS, inducible nitric oxide synthase; NOX, NADPH oxidase; HMGCR, 3-hydroxy-3-methylglutaryl coenzyme A reductase; RNR, ribonucleotide reductase; DHFR, dihydrofolate reductase; TRX, thioredoxin; PPP, pentose phosphate pathway; TLR4, Toll-like receptor 4; RNS, reactive nitrogen species; ROS, reactive oxygen species; NET, neutrophil extracellular trap.

Glucose-6-phosphate dehydrogenase (G6PD) catalyzes the rate-limiting first step in the pentose phosphate pathway (PPP), converting nicotinamide adenine dinucleotide phosphate (NADP) to its reduced form: NADPH (**Figure 1A**). Levels of active G6PD are regulated at both the transcriptional level via various canonical signaling pathways (e.g., JAK-STAT, Wnt, mTOR), and at the post-translational level (e.g., via phosphorylation, de-acetylation), allowing tight coordination of G6PD activity to meet acute cellular demand in response to oxidative stress, metabolic demand, or systemic inflammation (**Figure 1A-1**). NADPH generation is critical to host antioxidant defense via glutathione reduction (**Figure 1A-2**), and is also essential for anabolic cellular metabolism, including synthesis of nucleotides, fatty acids, and amino acids (**Figure 1A-3**). Downstream production of ribulose-5-phosphate (R5P) is essential for formation of key nucleotides and cofactors (**Figure 1A-3**), in addition to acting as a glycolytic shunt intermediary (**Figure 1A-4**). Indeed, G6PD, by virtue of its importance in fundamental redox homeostasis and anabolic metabolism, plays a multifaceted, ubiquitous role in human physiology, including within immune responses.

Across human populations, basal G6PD enzyme activity is highly variable, with hundreds of missense genetic mutations described worldwide in the X-linked *G6PD* gene. *G6PD* mutations lead to a range of enzyme deficiency phenotypes as classified by the World Health Organization, characterized by decreased enzyme stability and/or reduced G6PD enzyme activity. Indeed, G6PD deficiency is the most common human enzymopathy, affecting >500 million people worldwide. It has been hypothesized that G6PD deficiency confers a selective advantage against malaria, as there is substantial overlap of G6PD deficiency prevalence in regions of historical malaria-endemicity (1), although experimental evidence supporting this malaria protection hypothesis remains mixed (2–6). In the red blood cell (RBC), where G6PD deficiency has best been studied, and where the trait is most prominently manifested, oxidant stressors—e.g., infection, diet, and medications—can trigger life-threatening hemolytic crises and anemia. Beyond RBC biology and malaria, investigators have also examined the function of G6PD in immunity. Here, we review the role(s) of G6PD activity and the effects of G6PD deficiency on immune system function, in health and disease.

## 2 Importance of G6PD for innate immunity

G6PD and the PPP are crucial for innate immune system function. Key phagocytes, such as neutrophils and macrophages, use reactive oxygen species (ROS) and reactive nitrogen species (RNS) to kill pathogens in phagolysosomes. To generate sufficient quantities of ROS and RNS quickly, these cells require rapid flux through the PPP to produce sufficient amounts of NADPH to enable superoxide formation from oxygen by the action of NADPH oxidase (NOX), and nitric oxide (NO) formation from arginine by inducible nitric oxide synthase (iNOS) (**Figure 1A-2**). The required rapid flux through the PPP necessitates sufficient G6PD enzyme activity capacity to allow this respiratory burst to occur. Given that NOX and NOS activity in neutrophils and macrophages depend upon G6PD-derived NADPH and reduced glutathione (GSH) (7), the role of G6PD and the PPP has been studied in these cell types, and roles for G6PD activity have been identified even beyond the oxidative burst.

### 2.1 G6PD and PPP function in neutrophils

As the most common circulating blood leukocyte, neutrophils are crucial for monitoring pathogens and tissue damage. Soon after stimulation by an appropriate trigger—e.g., chemokines/cytokines, pathogen components like lipopolysaccharide (LPS), and/or wounded tissue—neutrophils migrate to areas of injury and/or infection and mount an initial innate immune response which requires a rapid metabolic switch dependent on G6PD. Minutes after initial stimulation, neutrophils switch from primarily using glycolysis for basal metabolism, to the PPP, in order to allow for rapid generation of NADPH (8).

Brisk production of NADPH through the PPP is itself linked to sufficient basal and reserve capacity of G6PD enzyme activity (**Figure 1A**). The NADPH generated allows for biosynthesis of effector molecules (**Figure 1A-3**), as well as rapid generation of ROS within phagolysosomes (**Figure 1A-2**). This was first demonstrated by finding that chemical inhibition of the neutrophil oxidative burst exhibited PPP and G6PD dependence (9). Indeed, several distinct stimuli, each with different receptors and mechanisms of action, have been found to induce the neutrophil respiratory burst and pentose cycling *ex vivo* (8). This G6PD-dependent rapid metabolic switch allows several crucial neutrophil activities to occur (**Figure 1B-1**): phagocytosis, release of neutrophil extracellular traps (NETs), and degranulation accompanied by rapid production of large amounts of extracellular ROS. Indeed, neutrophil activation and degranulation, including extrusion of NETs via NETosis, are all PPP- and ROS-dependent processes, generated by NOX activity (8, 10, 11).

In particular, neutrophil NETosis is a critical neutrophil effector function, which releases chromatin and granule proteins into the extracellular space to entangle and kill bacteria (12). In activated neutrophils, NET formation occurs in response to pathogen stimuli and pro-inflammatory signaling and depends on superoxide generation by NADPH oxidase (**Figure 1A-2**). The rapid production of ROS in activated neutrophils, which requires increased pentose cycling, and thus increased G6PD activity, acts to promote NETosis via oxidative inhibition of glyceraldehyde 3-phosphate dehydrogenase (GAPDH) (13); this process can even be pathogenic when excessive, as in hyperinflammatory states such as severe acute respiratory syndrome coronavirus 2 (SARS−CoV−2) infection (14).

Overall, the uniquely rapid need for neutrophil effector functions, such as NETosis, phagocytosis and degranulation, to be mobilized quickly in the innate immune response necessitates brisk production of NADPH, which is chiefly controlled by the PPP rate-limiting activity of G6PD.

### 2.2 G6PD function in macrophages

G6PD activity is also critical in macrophage function. Similar to neutrophils, rapid production of NADPH is needed for phagocytosis, and the oxidative burst in activated macrophages. Sufficient reserve capacity for G6PD activity allows for rapid production of excess NADPH needed with macrophage activation. This G6PD-dependent production of NADPH is then used for biosynthesis of effector molecules (**Figure 1A-3**), as well as ROS/RNS production (via NOX, iNOS) needed for phagocytosis and the oxidative burst (**Figure 1A-2**).

Beyond the phagocytic respiratory burst, G6PD acts more generally as a key regulator of cellular redox homeostasis and provides substrate for NADPH oxidase by generating NADPH, thus exerting an innate immune modulatory role in these capacities. While some ROS generation, produced via NADPH-dependent NOX and iNOS, is advantageous and necessary for pathogen clearance, it must be carefully titrated by antioxidant GSH – which is itself regulated by NADPH-dependent glutathione reductase; as both processes are NADPH-dependent, both processes depend on G6PD activity (**Figure 1A-2**). With respect to redox homeostasis, ROS induction has numerous downstream effects. For instance, excess ROS leads to activation of nuclear factor erythroid 2-related factor 2 (NRF2) and upregulation of heme oxygenase (HMOX), which can dampen inflammation and protect against immunopathology more generally. As an example, immunopathologic vasculopathy in a hypoxic model of pulmonary hypertension was found to be driven by G6PD-induced pro-inflammatory epigenetic signaling — including upregulated expression of pro-inflammatory factors (e.g., tumor necrosis factor-alpha [TNF-α]), increased numbers of activated macrophages, and activated platelets — and conversely, was able to be mitigated with G6PD deficiency (15, 16).

G6PD-dependent ROS generation is also important in innate immune sensing by macrophages (**Figure 1B-1**). Nucleotide-binding oligomerization domain (Nod)-like receptor (NLR) signaling via pattern recognition receptors also depends on PPP flux, which can affect intracellular sensing by macrophages of pathogens, such as *Aspergillus* (17). Priming the canonical NLR family pyrin domain containing 3 (NLRP3) inflammasome also depends on ROS. Indeed, G6PD-deficient monocytes exhibit decreased inflammasome responsiveness to LPS, including poor induction of pro-interleukin-1β (IL-1β), which has been linked to insufficient NADPH oxidase production of ROS and impaired intracellular bacterial killing (18). This impaired inflammasome responsiveness in G6PD deficiency may also in part explain the decreased body temperatures seen in G6PD-deficient patients with malaria (19).

Furthermore, systemic inflammatory response signals and their downstream propagation is dependent on G6PD expression and activity in macrophages (**Figure 1B-1**). As an example, in response to gram-negative LPS, TNF-α induces upregulation of macrophage G6PD expression and activity (20), and increased G6PD expression has been visualized by ultrastructural techniques in activated macrophages (21). By contrast, G6PD-deficient mice have a diminished innate response to LPS, with impaired IL-6 production (22). Macrophage-derived G6PD activity also links the innate and acquired immune system, with ROS upregulating immunoresponsive gene 1 (IRG1) and major histocompatibility complex I (MHC I) expression in activated macrophages in a PPP- and NOX-dependent manner (23).

### 2.3 Effect of G6PD deficiency on innate immune system function and disease

Given the role of G6PD in phagocyte effector function, in the 1960s, it was theorized that G6PD deficiency might impact susceptibility to sepsis and meningitis (24). In the most severe variants of G6PD deficiency, e.g., those leading to chronic non-spherocytic hemolytic anemia and chronic granulomatous disease (CGD), it was observed that leukocyte G6PD activity and PPP flux were markedly reduced (25, 26), leading to diminished oxidative burst and impaired bacterial killing in phagolysosomes (27). Moreover, in these sporadic cases of severe G6PD deficiency with CGD, PPP flux is markedly diminished to the point of impaired NADPH oxidase activity and absent NETosis (28), and the impaired phagocytic respiratory burst was clinically linked to cases of mycobacterial infection (29). Beyond the severe deficiency variants, it was also noted that there was reduced G6PD activity in leukocytes isolated from individuals with moderate G6PD deficiency such as those with the common Mediterranean variant (30–34).

Clinically, there is much interest in understanding the role that G6PD deficiency and an associated aberrant innate immune effector response might play in childhood sepsis. For example, G6PD activity is markedly diminished in neutrophils isolated from neonates, with variable reports of deficient phagocytosis and chemotaxis and impaired bactericidal killing (35, 36). Indeed, a case-control study of 76 neonates with sepsis and 1214 healthy neonates identified a significantly increased prevalence of G6PD deficiency in those neonates specifically admitted for sepsis (37). In G6PD-deficient infants in Saudi Arabia, where the Mediterranean variant predominates, an increased risk of severe infection and sepsis was seen in childhood, especially with catalase-positive organisms (38, 39). In Kenya, where the milder G6PD A- deficiency variant predominates, large case-control studies in children reported an increased risk for bacteremia, especially with gram positive organisms such as *Staphylococcus aureus* and *Streptococcus pneumoniae* (40, 41). Increased risk has also been reported for other intracellular pathogens, including *Salmonella typhi* [typhoid fever (42, 43)], *Rickettsia rickettsia* [Rocky Mountain spotted fever (44, 45)], and *Toxoplasma gondii* [toxoplasmosis (46)]. The added impact of G6PD deficiency may be even more pronounced in settings of more severe immune compromise; for example, patients with acute myelogenous leukemia and concomitant G6PD deficiency exhibit increased risk for invasive fungal infections (47).

Apart from serious bacterial infection, other stressors can activate a brisk systemic innate immune response, including trauma and severe coronavirus disease 2019 (COVID-19). In one study of African-American trauma patients, those with G6PD A- deficiency were at increased risk for prolonged systemic inflammatory response syndrome, septicemia, and respiratory and wound infections (48). The hyperinflammation associated with severe COVID-19 infection has been linked to an overly exuberant innate immune phenotype with excessive macrophage activation (49) that could plausibly be impacted by G6PD deficiency. While genome-wide association studies of COVID-19 have not specifically linked *G6PD* polymorphic variants to severe illness (50), several retrospective studies have suggested that patients with G6PD deficiency were at increased risk of hospitalization and severe disease (51, 52), including acute respiratory distress syndrome with associated ventilator dependence (53).

## 3 G6PD in adaptive immunity

Adaptive immunity, specifically the effector arms of T and B cell activation, are also dependent on G6PD and PPP activity. Over 50 years ago, it was first noted that G6PD activity increases in lymphocytes activated by phytohemagglutinin [PHA] (54). More recently, several groups interested in cancer biology have detailed a variety of cell-type specific lymphocyte functions that are impacted by G6PD. In particular, these recent efforts have shown that activated T cells depend on G6PD and PPP activity for fundamental processes related to effector function, ranging from metabolic reprogramming and ROS homeostasis, to biosynthesis of effector molecules and signal transduction (**Figure 1B-2**).

In activated T cells, metabolic fitness and synthetic function has been directly linked to G6PD activity and ROS homeostasis by Ghergurovich and colleagues, who used a novel metabolite reporter and deuterium tracer assays to monitor cellular G6PD activity in the presence of a specific potent inhibitor of G6PD (55). In activated T cells, flux through the oxidative PPP increases nearly 10-fold, but chemical inhibition of G6PD can markedly decrease this and, as a result, impair T cell activation and decrease production of pro-inflammatory interferon-gamma (IFN-γ) (55). This dependence on G6PD activity in activated T cells occurs despite the partially redundant contribution of other enzymes like isocitrate dehydrogenase and malate dehydrogenase to the overall production of cellular NADPH, which is needed for biosynthesis of immune effector molecules (**Figure 1A-3**). In addition, G6PD has a critical role, along with NFKB-inducing kinase (NIK), in supporting metabolism in activated T cells (56). In particular, activated T cells maintain aerobic glycolytic flux by stabilizing hexokinase 2, which requires control of ROS via G6PD and the PPP (**Figure 1A-2**). G6PD-deficient activated T cells exhibit diminished glycolytic metabolism and reduced cytokine production (55), and cytokine-induced killer cells likewise depend on the PPP for effector function and proliferation (57).

Beyond metabolism, G6PD activity plays a role in T cell signal transduction and cytolytic function (**Figure 1B-2**). In early T cell receptor activation, intracellular calcium signaling is dependent on G6PD and dual oxidase 1 (DUOX1) enzyme activity, which work in concert to facilitate NADP/NADPH cycling (58). In cytotoxic T lymphocytes, G6PD activity has also been linked epigenetically to granzyme B production and cellular lytic function via acetyl coenzyme A synthesis and histone acetylation (59).

ROS homeostasis, which is highly dependent on G6PD activity (**Figure 1A-2**), is also critical for T cell function, including maturation and activation (60). In particular, ROS affects T cell receptor signaling through several downstream signaling pathways (57, 61, 62), including mitogen-activated protein kinase (MAPK), phosphoinositide 3-kinases (PI3K), and Janus kinase-signal transducer and activator of transcription 3 (JAK-STAT3). Some amount of ROS is critical for normal T cell survival and maturation, and appropriate physiologic upregulation of ROS levels is needed for T cell receptor activation and downstream signaling that leads to T helper cell type 1 (Th1) differentiation and production of pro-inflammatory cytokines, such as IFN-γ and TNF-α (63). However, too much ROS can lead to excess Th1 activation and promote a pro-inflammatory state. Indeed, in high metabolic states, T cells depend on higher levels of G6PD activity to maintain homeostasis by mitigating ROS. For example, in T-lymphoblastic leukemia cells, inhibition of mammalian target of rapamycin (mTOR) signaling downregulates G6PD, resulting in excess ROS and cell death (64).

In B cells, basal G6PD expression and activity are low, suppressed by transcription factors paired box protein 5 (PAX5) and Ikaros family zinc finger protein 1 (IKZF1). However, in states of activation, including oncogenesis, B cell activation depends on G6PD and PPP activity to mitigate oxidative stress (65), and the hypermetabolic leukocytes of acute B-cell leukemia are likewise more sensitive to oxidant stressors, such as dihydroartemisinin (66).

## 4 The effect of G6PD in other cell types important for immunity

Beyond leukocytes, it is relevant to consider other cell types that contribute to innate immune activation and systemic inflammatory responses, including red blood cells, platelets, hepatocytes, and endothelial cells. These cell types are also impacted by G6PD and PPP activity with respect to ROS homeostasis and biosynthesis of effector molecules, and this can impact their downstream contribution to systemic inflammation.

### 4.1 Red blood cells

G6PD is a key regulator of oxidant stress in erythrocytes, critical to protection against membrane stress and hemolysis, which is a potent systemic inflammatory stimulus (**Figure 1B-3**). For example, free heme triggers macrophage Toll like receptor 4 (TLR4) activation and TNF-α production, as well as ROS-mediated NLRP3 inflammasome induction (67–70). Additionally, increased serum iron levels caused by frequent hemolysis may play a role in immune modulation, as several studies have linked hyperferremia with serious infection in humans, a concept termed “nutritional immunity” (71–74). Beyond hemolysis, recent evidence suggests a role for microvesicle formation and mitochondrial retention in oxidatively-stressed RBCs in potentiating immune activation (75, 76). Such oxidatively stressed RBCs then serve to facilitate macrophage activation by binding macrophage Fc receptors; indeed, with respect to malaria protection, it has been theorized that protection conferred by G6PD deficiency may be related to increased phagocytosis of parasitized oxidatively-damaged G6PD-deficient RBCs (77–79) via binding by macrophage Fcγ and lectin-like receptors (80), and that such parasites may be more susceptible to ROS (81).

### 4.2 Platelets

Platelets are another key participant in the innate immune response. They act as sensors, mediators, and direct effectors, and platelets promote endothelial activation, leukocyte recruitment, systemic inflammation, and pathogen neutralization. Platelets recruit and activate cells in both innate and adaptive immune responses and also secrete antimicrobial peptides (82, 83). Platelets directly sense pathogen- and damage-associated signals via TLRs and other receptors (84). Platelets also possess complement and cytokine/chemokine receptors, in addition to producing and releasing pro-inflammatory cytokines upon activation (85). Once activated, platelets secrete various immunomodulatory and microbicidal factors stored in their alpha-granules, including defensins, thrombocidins, kinocidins, and other antimicrobial peptides (85). Platelets can bind and be activated by damage and pathogen signals, including viral particles and bacterial surface proteins (86); in addition, platelet degranulation can promote direct bactericidal activity and enhance macrophage clearance (87). As a testament to their key roles in infection and systemic inflammation, it is noteworthy that pro-inflammatory cytokines, such as IL-6, regulate platelet production (88).

Thus, although platelets are less well studied in G6PD deficiency, they are also relevant for immune function (**Figure 1B-4**). As an example, platelets from G6PD-deficient individuals exhibit reduced G6PD enzymatic activity (30), affecting the availability of reduced glutathione required for platelet aggregation (89) and of NADPH needed for biosynthesis of fatty acids and phospholipids (90) (**Figure 1A-3**). Additionally, activated platelets require a switch to aerobic glycolysis, which increases PPP flux; a process that can be abrogated via chemical inhibition of G6PD (91).

### 4.3 Liver

G6PD function in the liver is relevant to systemic immunity, given the liver’s key role in producing relevant plasma proteins, including acute phase reactants, cytokines, and complement factors (**Figure 1B-5**). The liver also amplifies innate immune activation by producing mediators, such as IL-6 and C-reactive protein (CRP), and liver dysfunction results in a relative immunodeficient state (92). Hepatic G6PD enzymatic activity is diminished in G6PD-deficient individuals (93), can be altered in non-deficient individuals by exogenous factors, such as insulin and high fat diets (94, 95), and can be upregulated to meet increased demand in states of physiologic stress. For example, in G6PD-deficient individuals, residual hepatic enzyme activity can prove insufficient in stress states where increased G6PD activity is required, as in neonates with comorbid G6PD deficiency and Gilbert’s syndrome, who are at increased risk for hyperbilirubinemia (96, 97). As a corollary, in response to oxidant stress, G6PD-deficient hepatocytes exhibit transcriptomic network perturbations in redox pathways and immune response pathways (98). Clinically, G6PD deficiency has been linked to increased risk of Hepatitis A and B infections (99, 100), which may be related to deficient interferon responses in hepatocytes due to insufficient ROS generation by NADPH oxidase, as has been described for Dengue virus infection in G6PD-deficient monocytes (101).

### 4.4 Endothelium and epithelium

Tissue barrier cells, including epithelial and endothelial cells, also play key roles in host-pathogen interactions and immunopathology (**Figure 1B-6**). In G6PD-deficient individuals, epithelial and mesenchymal cells such as fibroblasts and lenses, exhibit decreased enzyme activity (102, 103). In the endothelium, G6PD activity plays a key role in mediating endothelial cell homeostasis and VEGF-mediated angiogenesis (104), as depletion of enzyme activity is associated with decreased endothelial NOS activity and decreased NO bioavailability. In response to systemic inflammatory factors (e.g., ROS, LPS-mediated TLR4 signaling, TNF-α), G6PD undergoes compensatory upregulation in the endothelium (20, 105). This is likely to be an issue in G6PD-deficient individuals because an inability to maintain endothelial homeostasis has been linked to both acute and chronic inflammatory endothelial activation and has relevance to pathologies as diverse as cardiovascular disease and sepsis. Less well studied, but equally intriguing, is the effect of G6PD deficiency on airway epithelial barrier function. For example, G6PD activity and PPP flux were found to be essential for innate immune sensing of *Aspergillus* infection by Nod-like receptors in airway epithelial cells (17).

## 5 Discussion

The G6PD story is one of variance: just as inherited levels of enzymatic activity vary across the geographic spectrum, so too do they vary across the spectrum of health and disease. G6PD is an acutely responsive sensor of cellular and metabolic stress, which is modulated by a myriad of factors, including diet and environment. In leukocytes, G6PD activity exhibits diurnal variation (106), is reduced in states of malnutrition (107), and is upregulated during bacterial infection (108). Beyond infection, G6PD may also play a role in the immunopathology of autoimmune disease. As examples, emerging evidence links increased G6PD activity and PPP flux to hyper-functional dendritic cells in aplastic anemia (109), as well as pro-inflammatory activated T cells in rheumatoid arthritis (110); still other reports link G6PD deficiency to a range of immune phenotypes (111), including celiac disease (112) and asthma (113). Finally, G6PD may play a role in immunosenescence, given that G6PD activity in lymphocytes declines with age (114, 115).

Indeed, given the ubiquity of G6PD in immunologically-relevant pathways and processes, it is clear that more research is needed to improve our understanding of the immunologic role of G6PD and G6PD deficiency in health and disease. At a macroscopic level, by considering the population genetics of the world’s most common enzymopathy, it is worth asking anew what the beneficial and harmful roles of G6PD deficiency are in immune function that might affect evolutionary fitness in the face of infectious and environmental stressors. Common functional variants at *G6PD* have independently arisen on diverse genetic backgrounds across a wide geographic range and exhibit variable penetrance with respect to enzyme activity; thus, the resulting immunologic phenotypes also likely vary considerably across populations. How might such variation differentially affect cellular and immune homeostasis and the body’s response to immunopathologic stressors, including innate and adaptive immune responses to severe infection? Could G6PD play a role in response to vaccination or in predispositions to autoimmunity? Ideally, answers to such questions will help paint a clearer picture of the role of this interesting enzyme in immune function and immunopathology, and might someday allow physicians to harness immunometabolic diagnostic and therapeutic tools to improve outcomes in severe sepsis and various immune disorders.

## Author contributions

SS: Writing – original draft, Writing – review & editing. ES: Writing – original draft, Writing – review & editing. RF: Writing – original draft, Writing – review & editing. MK: Writing – original draft, Writing – review & editing.
